# The synthesis of axially disubstituted silicon phthalocyanines, their quaternized derivatives and first inhibitory effect on human cytosolic carbonic anhydrase isozymes hCA I and II

**DOI:** 10.1039/c7ra13674a

**Published:** 2018-03-13

**Authors:** Tayfun Arslan, Zekeriya Biyiklioglu, Murat Şentürk

**Affiliations:** Department of Chemistry, Faculty of Sciences 28200 Giresun Turkey tayfunars28@hotmail.com +90 454 310 15 04; Department of Textile, Technical Sciences Vocational School, Giresun University 28049 Giresun Turkey tayfunars28@hotmail.com +90 454 310 15 04; Department of Chemistry, Faculty of Science, Karadeniz Technical University 61080 Trabzon Turkey zekeriya_61@yahoo.com +90 462 377 36 64; Department of Basic Sciences of Pharmacy, Faculty of Pharmacy, Agri Ibrahim Cecen University 04100 Agri Turkey senturkm36@gmail.com +90 0472 215 98 63

## Abstract

In this study a novel silicon(iv) phthalocyanine bearing [(2*E*)-3-[4-(dimethylamino)phenyl]-1-(4-phenoxy)prop-2-en-1-one] group and its quaternized derivative at their axial positions were synthesized for the first time. Axially disubstituted silicon(iv) phthalocyanines were also characterized by various spectroscopic techniques. The inhibition of two human cytosolic carbonic anhydrase (hCA, EC 4.2.1.1) isozymes I and II, with axially disubstituted silicon phthalocyanines and their quaternized derivatives were investigated by using the esterase assay, with 4-nitrophenyl acetate as substrate. Silicon phthalocyanines ZM-1-Si, ZM-5-Si, ZT-Si and their quaternized derivatives ZM-1-SiQ, ZM-5-SiQ, ZT-SiQ showed IC_50_ values in the range of 0.0178–0.1653 μM for hCA I and of 0.0172–0.1212 μM against hCA II, respectively. This study is the first example of carbonic anhydrase enzyme inhibition of phthalocyanines.

## Abstract 2

In this study a novel silicon(iv) phthalocyanine bearing [(2*E*)-3-[4-(dimethylamino)phenyl]-1-(4-phenoxy)prop-2-en-1-one] group and its quaternized derivative at their axial positions were synthesized for the first time.

## Graphical abstract

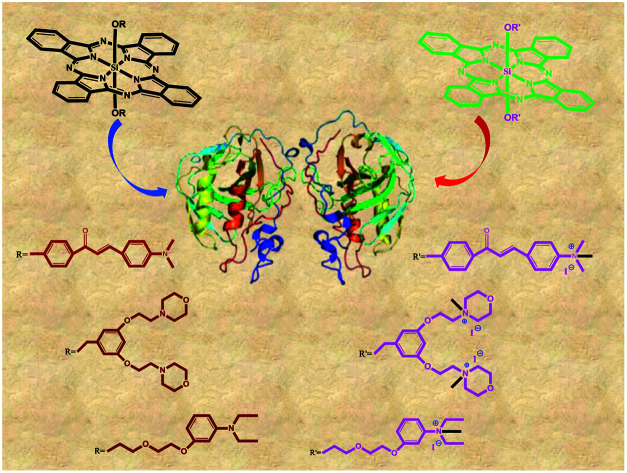

## 1. Introduction

Carbonic anhydrase (EC 4.2.1.1, CA) is a metalloenzymes family that catalyzes the rapid conversion of CO_2_ to HCO_3_^−^ and H^+^.^1^ CA isoforms are found in a variety of tissues where they participate in several important biological processes such as acid–base balance, respiration, carbon dioxide and ion transport, bone resorption, ureagenesis, gluconeogenesis, lipogenesis and electrolyte secretion.^2–6^ Many CA isozymes involved in these processes are important therapeutic targets with the potential to be inhibited/activated for the treatment of a range of disorders such as edema, glaucoma, obesity, cancer, epilepsy and osteoporosis.^2,4^ Our groups recently investigated the interaction of 12 mammalian CA isozymes with several types of phenolic compounds, such as catechol and a series of phenols and phenolic acids, *e.g.*, catechol, resorcinol, salicyclates and some of their derivatives. They are reported to possess anticancer, anti-carcinogenic, antimutagenic, antibacterial, antiviral or anti-inflammatory activities. Phenol, phenolic compounds and hydroxybenzoic acid derivatives are widely used prodrugs or drugs. Salicylic acid is known for its ability to ease aches and pains and reduce fevers. These medicinal properties, particularly fever relief, have been known since ancient times, and it was used as an anti-inflammatory drug.^7–9^

Indeed, phenol binds to CA in a diverse manner compared to the classical inhibitors of the sulfonamides/sulfamates/sulfamides, which coordinate to the Zn^2+^ ion from the enzyme active site by substituting the fourth, non-protein ligand, a water molecule or hydroxide ion.^10^ Recently, Christianson's group then reported the X-ray crystal structure for the adduct of hCA II with phenol, showing indeed this inhibitor to bind to hCA II by anchoring its OH moiety to the zinc-bound H_2_O/hydroxide ion of the enzyme through a hydrogen bond as well as to the NH amide of Thr 199, an amino acid conserved in all α-CAs and critically important for the catalytic cycle of these enzymes. Furthermore, the phenyl moiety of this inhibitor was found to lay in the hydrophobic part of the hCA II active site, where presumably CO_2_, the physiologic substrate of the CAs, binds in the precatalytic complex, explaining thus the behaviour of phenol as a unique CO_2_ competitive inhibitor.^10^

**Fig. 1:**
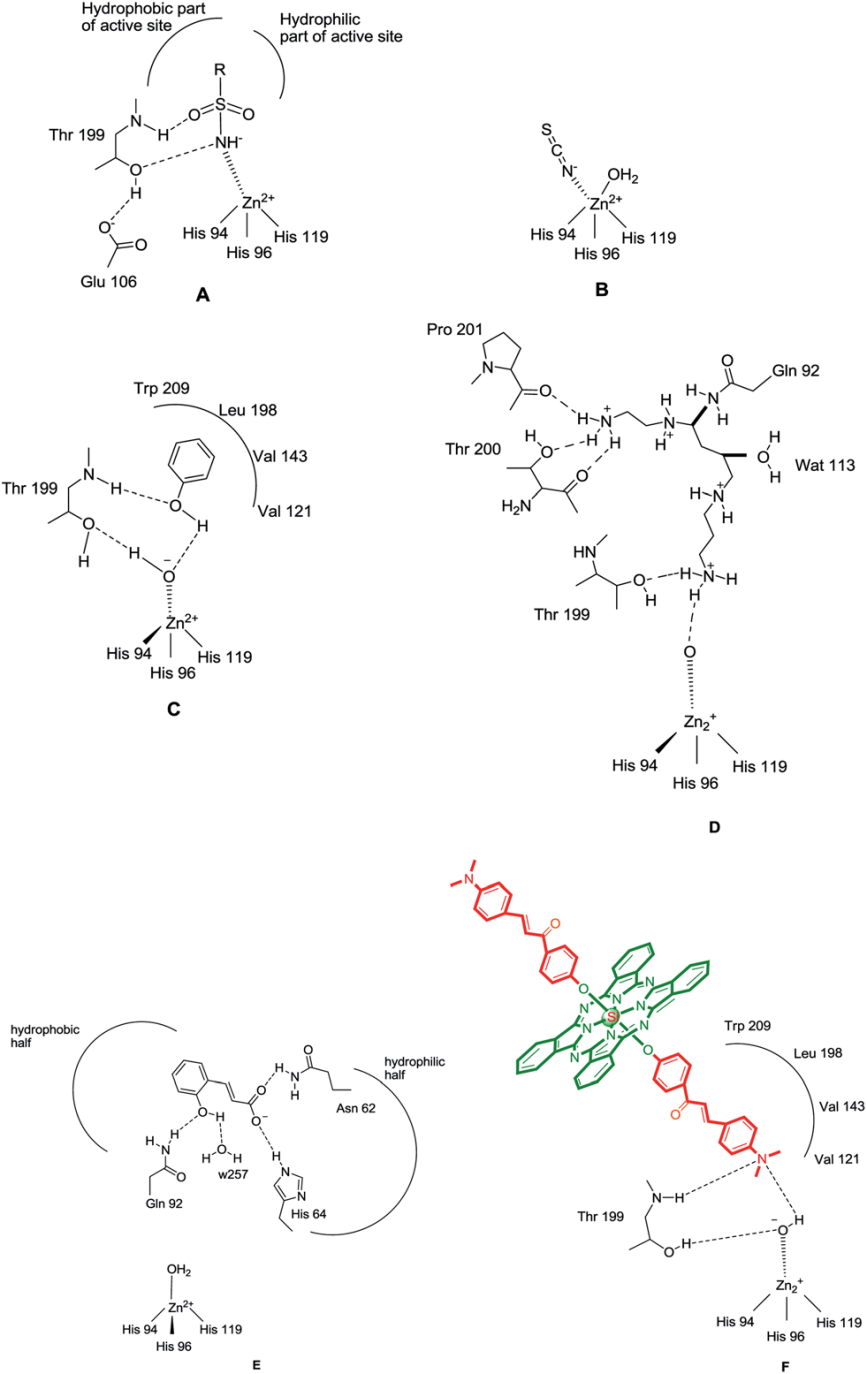
CA inhibition with: zinc binders such as sulfonamides (A) and inorganic anions (B); compounds anchoring to the zinc-bound water/hydroxide ion, such as phenol (C), spermine (D) and compounds occluding the entrance to the CA active site cavity, exemplified by the hydrolyzed coumarin, *trans*-2-hydroxycinnamic acid (E). Figures represent distances (in Å), as determined by X-ray crystallographic techniques.^10,12^ (F) Hydrogen bonds are represented as dashed lines. All these binding modes have been proven by means of X-ray crystallography on enzyme-inhibitor adducts.^10^

The CAIs belong to four main classes: (i) sulfonamides (and their isosteres, such as sulfamates, sulfamides and similar derivatives) and metal complexing anions, which coordinate to the Zn(ii) ion from the enzyme active site in tetrahedral or trigonal bipyramidal geometries of the metal ion (Fig. 1A and B),^11^ (ii) phenols (such as the simple phenol C_6_H_5_OH),^10,12^ which bind to the zinc-coordinated water molecule/hydroxide ion from the active site, through a network of two hydrogen bonds (Fig. 1C), (iii) the polyamines,^11^ such as spermine, spermidine and congeners, which bind rather similar but not identical to phenols, that is, by anchoring to the water molecule/hydroxide ion coordinated to Zn(ii), Fig. 1D and (iv) the recently reported class of effective CAIs, the coumarins and thiocoumarins, which have an inhibition mechanism not dependent of Zn(ii), and bind (in hydrolyzed form) in the same active site region as the activators, occluding the entrance to the active site (Fig. 1E).^12^

Phthalocyanines (Pcs) in the family of dyes, are well-known planar compounds with highly fluorescent, very good thermal and chemical stability.^13^ Because of these properties of Pcs dyes have been attracting increasing interest. Pcs dyes have found their roles in numerous fluorescent materials and photodynamic therapy applications. Such dyes and their derivatives widespreadly have been used in different technological areas such as liquid crystals, electronic devices, gas and chemical sensors, electrochromic and electroluminescent displays, non-linear optics, photovoltaics, semiconductors, photodynamic therapy and so forth.^14^ The low solubility of phthalocyanines in organic and water solvents and their aggregation is an important problem in biological application of phthalocyanines. To overcome this problem, the introduction of substituents at the axial positions of phthalocyanines is preferred because the axial positions can strongly influence some properties of phthalocyanines such as its solubility and aggregation behavior.^15,16^ For this reason, non-aggregating and water soluble axially disubstituted silicon phthalocyanines (SiPcs) can be used in biological applications. Previous researches have also demonstrated that toxicity of SiPcs are very low against cancer cells.^17,18^

But, researches on biological study of SiPcs are quite limited in literature.^19–25^ According to our knowledge, the carbonic anhydrase inhibitor properties of silicon phthalocyanines has not been reported in the literature. We report herein, the synthesis and characterization of the novel silicon(iv) phthalocyanine bearing [(2*E*)-3-[4-(dimethylamino)phenyl]-1-(4-phenoxy)prop-2-en-1-one] group and its quaternized. Also their human cytosolic carbonic anhydrase isozymes hCA I and II inhibitory properties were investigated for the first time.

## 2. Experimental section

### 2.1. Materials and methods

All reagents and solvents were of reagent grade quality and were obtained from commercial suppliers. All solvents were dried and purified as described by Perrin and Armarego.^26^ Sulphanilamide, Sepharose 4B, protein assay reagents, 4-nitrophenylacetate were obtained from Sigma-Aldrich Co. All other chemicals were analytical grade and obtained from Merck.

The IR spectra were recorded on a Perkin Elmer 1600 FT-IR spectrophotometer, using KBr pellets. ^1^H and ^13^C-NMR spectra were recorded on a Bruker Avance III 400 MHz spectrometers in CDCl_3_ and chemical shifts were reported (*δ*) relative to Me_4_Si as internal standard. MALDI-MS of complexes were obtained in dihydroxybenzoic acid as the MALDI matrix, using a nitrogen laser accumulating 50 laser shots, with a Bruker Microflex LT MALDI-TOF mass spectrometer. Optical spectra in the UV-Vis region were recorded with a Perkin Elmer Lambda 25 spectrophotometer.

### 2.2. Synthesis

#### 2.2.1. Bis[(2*E*)-3-[4-(dimethylamino)phenyl]-1-(4-phenoxy)prop-2-en-1-one]phthalocyaninato silicon(iv) (ZT-Si)

A mixture of SiPcCl_2_ (1) (100 mg, 0.16 mmol) and (2*E*)-3-[4-(dimethylamino)phenyl]-1-(4-hydroxyphenyl)prop-2-en-1-one (2) (85 mg, 0.32 mmol) in toluene (10 mL) was stirred and then sodium hydride (7.7 mg, 0.32 mmol) was added to this mixture. After heating at reflux temperature under nitrogen atmosphere for 24 h, toluene was evaporated to dry under reduced pressure. The green product was purified by column chromatography [silica gel/CHCl_3_ : CH_3_OH (100 : 6)]. Yield: 61 mg (35%). FT-IR (KBr pellet) *ν* (cm^−1^): 3021 (Ar–H), 2984–2848 (Aliph. C–H), 1645, 1579, 1550, 1503, 1430, 1334, 1289, 1263, 1210, 1160, 1120, 1079, 1038, 912, 881, 759, 729, 680. ^1^H-NMR (400 MHz, DMSO-d_6_), (*δ*:ppm): 9.74–9.72 (m, 8H, Pc–H_α_), 8.58–8.55 (m, 12H, Ar–H), 8.31–8.28 (m, 8H, Pc–H_β_), 7.68–7.64 (m, 4H, –CH=), 6.86 (m, 2H, Ar–H), 6.75 (m, 2H, Ar–H), 2.99 (s, 12H, CH_3_–N). ^13^C-NMR (100 MHz, DMSO-d_*6*_), (*δ*:ppm): 187.65, 160.23, 158.22, 149.48, 145.88, 136.04, 135.34, 132.22, 130.54, 129.41, 128.76, 124.96, 122.34, 118.62, 110.96, 40.34. UV-Vis (DMF) *λ*_max_ nm (log *ε*): 683 (4.97), 650 (4.26), 614 (4.31), 405 (4.50), 354 (4.59). MALDI-TOF-MS *m*/*z* calc. for C_66_H_48_N_10_O_4_Si 1073.23; found: 1074.24 [M + H]^+^.

#### 2.2.2. Axially bis[(2*E*)-3-[4-(dimethylamino)phenyl]-1-(4-phenoxy)prop-2-en-1-one]phthalocyaninato silicon(iv) iodide (ZT-SiQ)

A mixture of bis[(2*E*)-3-[4-(dimethylamino)phenyl]-1-(4-phenoxy)prop-2-en-1-one]phthalocyaninato silicon(iv) (ZT-Si) (30 mg, 0.028 mmol), 3.5 mL of methyl iodide in 4 mL chloroform was stirred at room temperature for 3 days. The green precipitate was filtered off, washed with chloroform and diethyl ether. Quaternized silicon phthalocyanine ZT-SiQ was dried *in vacuo*. Yield: 15 mg (38%). FT-IR (KBr pellet) *ν* (cm^−1^): 3042 (Ar–H), 2964–2893 (Aliph. C–H), 1650, 1591, 1523, 1502, 1429, 1335, 1211, 1160, 1120, 1027, 911, 882, 805, 733, 671. UV-Vis (DMF) *λ*_max_ nm (log *ε*): 684 (4.99), 614 (4.31), 414 (4.32), 355 (4.65), 324 (4.66). MALDI-TOF-MS *m*/*z* calc. for C_68_H_54_I_2_N_10_O_4_Si 1357.11; found: 1103.08 [M − 2I]^+^.

### 2.3. Biological studies

#### 2.3.1. Purification of carbonic anhydrase isozymes I and II from human erythrocytes by affinity chromatography

Erythrocytes were purified from fresh human blood obtained from the Blood Centre of the Research Hospital at Atatürk University. The blood samples were centrifuged at 1500 rpm for 15 min and the plasma and buffy coat were removed. The red cells were isolated and washed twice with 0.9% NaCl, and hemolyzed with 1.5 volumes of ice-cold water. The ghost and intact cells were removed by centrifugation at 20 000 rpm for 30 min at 4 °C. The pH of the hemolysate was adjusted to 8.7 with solid Tris.^27^ The hemolysate was applied to the prepared Sepharose 4B-aniline-sulfanilamide affinity column equilibrated with 25 mM Tris–HCl/0.1 M Na_2_SO_4_ (pH 8.7). The affinity gel was washed with 25 mM Tris–HCl/22 mM Na_2_SO_4_ (pH 8.7). The human carbonic anhydrase (hCA I and hCA II) isozymes were eluted with 1 M NaCl/25 mM Na_2_HPO_4_ (pH 6.3) and 0.1 M CH_3_COONa/0.5 M NaClO_4_ (pH 5.6), respectively. All procedures were performed at 4 °C.^27^

#### 2.3.2. Esterase activity assay

Carbonic anhydrase activity was assayed by following the change in absorbance at 348 nm of 4-nitrophenylacetate (NPA) to 4-nitrophenylate ion over a period of 3 min at 25 °C using a spectrophotometer (Shimadzu UV-1800) according to the method described by Verpoorte *et al.*^28^ The enzymatic reaction, in a total volume of 3.0 mL, contained 1.4 mL 0.05 M Tris–SO_4_ buffer (pH 7.4), 1 mL 3 mM 4-nitrophenylacetate, 0.5 mL H_2_O and 0.1 mL enzyme solution. A reference measurement was obtained by preparing the same cuvette without enzyme solution. The inhibitory effects of compounds were examined. All compounds were tested in triplicate at each concentration used. Different inhibitor concentrations were used. Control cuvette activity in the absence of inhibitor was taken as 100%. For each inhibitor an activity (%)–[inhibitor] graphs were drawn.^29^

## 3. Results and discussion

### 3.1. Synthesis and characterization

**Fig. 2:**
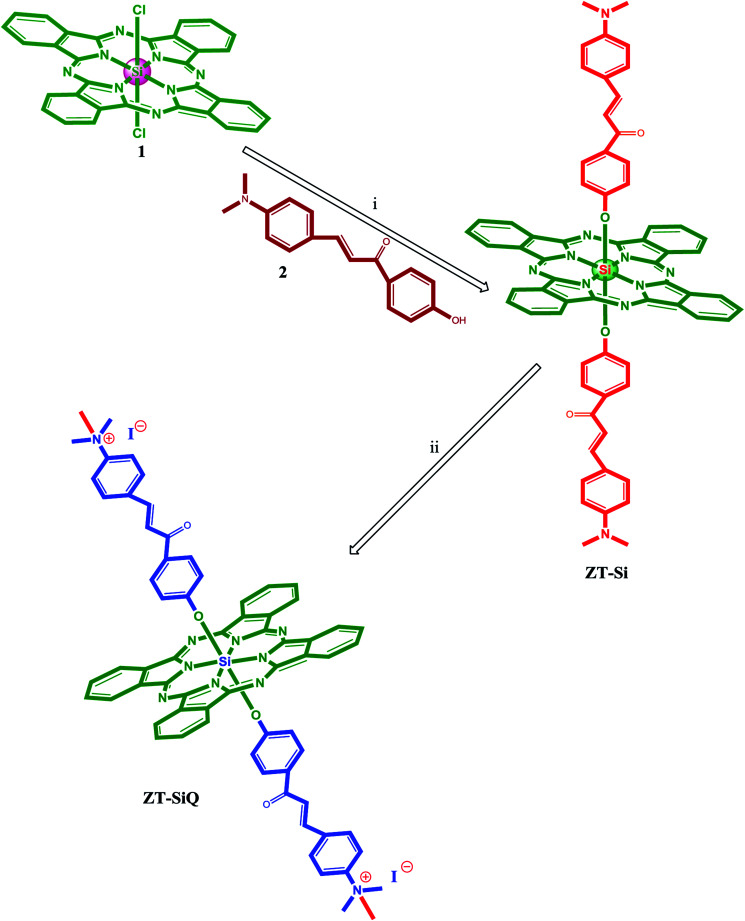
The synthesis of the silicon(iv) phthalocyanine ZT-Si and its quaternized derivative ZT-SiQ. (i) Toluene, NaH, reflux. (ii) CHCl_3_, CH_3_–I, room temperature.

**Fig. 3:**
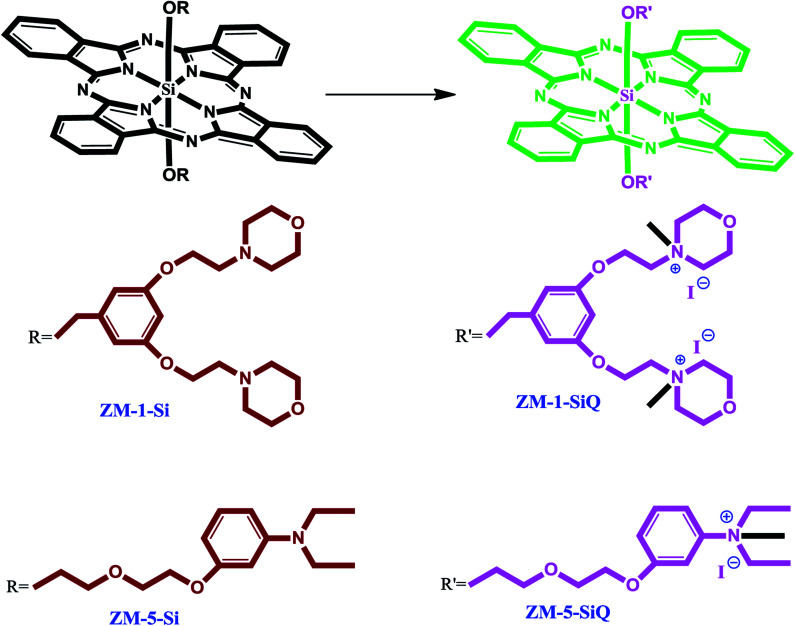
The synthesis of the silicon(iv) phthalocyanines ZM-1-Si, ZM-5-Si and their quaternized derivative ZM-1-SiQ, ZM-5-SiQ.

The synthesis of axially disubstituted silicon phthalocyanines ZT-Si, ZM-1-Si, ZM-5-Si and their quaternized derivatives ZT-SiQ, ZM-1-SiQ, ZM-5-SiQ were given in Fig. 2 and 3, respectively. (2*E*)-3-[4-(Dimethylamino)phenyl]-1-(4-hydroxyphenyl)prop-2-en-1-one 2,^30^ silicon(iv) phthalocyanine dichloride 1,^31^ silicon(iv) phthalocyanine ZM-1-Si, ZM-1-SiQ^32^ and silicon(iv) phthalocyanine ZM-5-Si, ZM-5-SiQ^33^ were synthesized according to previously published methods. Reaction of silicon(iv) phthalocyanine dichloride 1 with (2*E*)-3-[4-(dimethylamino)phenyl]-1-(4-hydroxyphenyl)prop-2-en-1-one 2 in the present of NaH in toluene led to the target axially disubstituted silicon(iv) phthalocyanine ZT-Si yielded 35%. Quaternized silicon(iv) phthalocyanine ZT-SiQ was achieved by the reaction of silicon(iv) phthalocyanine ZT-Si with excess methyl iodide which is a quaternization agent in CHCl_3_ at room temperature. The newly synthesized silicon(iv) phthalocyanine ZT-Si and its quaternized derivative ZT-SiQ were characterized by various spectroscopic methods including FT-IR, ^1^H NMR, ^13^C NMR, UV-Vis, mass. All the results were consistent with the predicted structures for all newly phthalocyanines as shown in the Experimental section.

**Fig. 4:**
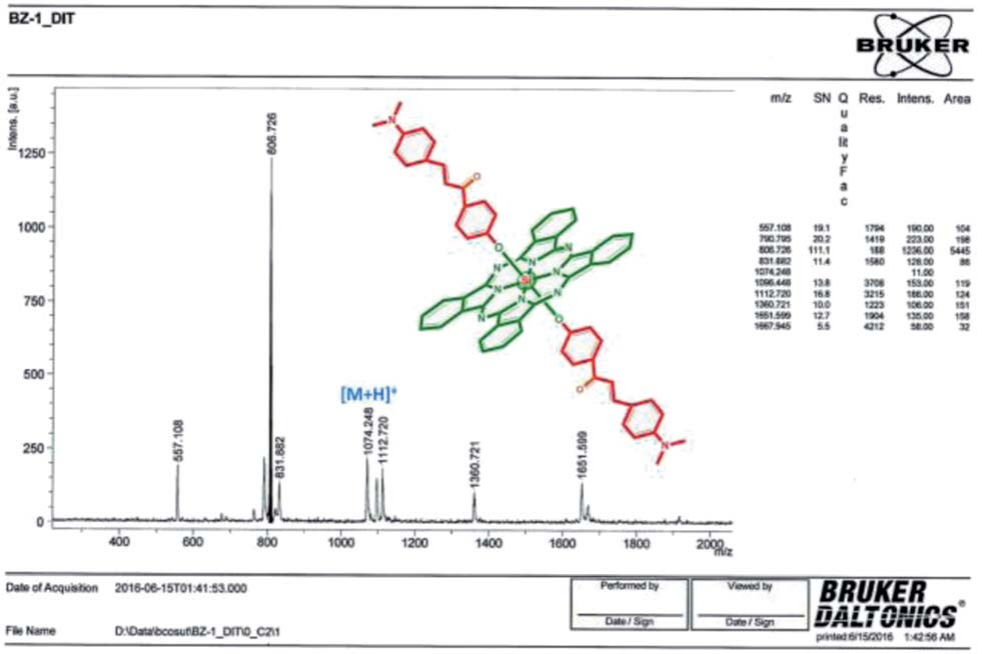
MALDI-TOF mass spectrum of ZT-Si.

The formation of silicon(iv) phthalocyanine ZT-Si was clearly confirmed by the disappearance of the OH band at 3318 cm^−1^ for compound 2 in the IR spectrum of phthalocyanine ZT-Si. The ^1^H NMR spectrum of axially disubstituted silicon(iv) phthalocyanine ZT-Si showed peaks belonging to H_α_ and H_β_ protons at between 9.74–9.72 and 8.58–8.55 ppm, respectively. In the ^1^H-NMR spectra of silicon(iv) phthalocyanine ZT-Si, the observation of new signals at *δ* = 8.31, 6.86, 6.75 ppm belonging to aromatic protons on the substituents proved the synthesis of this phthalocyanine ZT-Si. On the other hand, the appearance of new signal at *δ* = 2.99 ppm belonging to aliphatic protons (CH_3_–N) also confirmed the formation of target compound. The ^13^C-NMR spectra showed signals for relative carbon atoms for silicon(iv) phthalocyanine ZT-Si. The mass spectra of silicon(iv) phthalocyanine ZT-Si also confirmed the proposed structures of this phthalocyanine. The molecular ion peak was observed at *m*/*z*: 1074 as [M + H]^+^ (Fig. 4). No major change in the IR spectra was also observed after quaternization (for ZT-SiQ) of silicon(iv) phthalocyanine ZT-Si. The fragment peaks was observed to the mass spectra of quaternized cationic silicon(iv) phthalocyanine ZT-SiQ at *m*/*z*: 1103 as [M − 2I]^+^. This result support the proposed formula for silicon(iv) phthalocyanine ZT-SiQ.

**Fig. 5:**
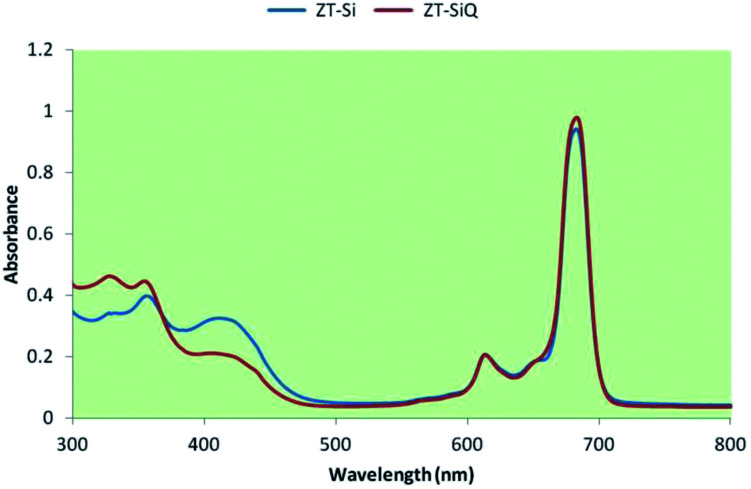
UV-Vis spectrum of ZT-Si and ZT-SiQ in DMF.

The ground state electronic absorption spectra of the novel non-ionic silicon(iv) phthalocyanine ZT-Si showed characteristic absorptions in the Q band region at 683 in DMF. The methyl group on the nitrogen atom of the substituents did not any affect on the absorption wavelengths of the studied phthalocyanine. The B band absorption of silicon(iv) phthalocyanine ZT-Si was observed at 405 and 354 nm (Fig. 5). The ground state electronic spectra of the quarternized silicon phthalocyanine ZT-SiQ showed characteristic absorption in the Q band region at 684 nm in DMF (Fig. 5). The quaternization of the non-ionic phthalocyanines did not affect to the absorption wavelength of the studied phthalocyanines. The B bands were observed at 414, 355 and 324 nm which are similar wavelength with non-ionic phthalocyanine ZT-Si in DMF.

### 3.2. Biological evaluation of the synthesized and reference compounds for CA inhibitory activity

The purification of the two CA isozymes used here was performed with a simple one step method by a affinity chromatography.^34^ Inhibitory effects of silicon phthalocyanines ZM-1-Si, ZM-5-Si, ZT-Si and their quaternized derivatives ZM-1-SiQ, ZM-5-SiQ, ZT-SiQ on enzyme activities were tested for the first time under *in vitro* conditions; IC_50_ values are given in Table 1.

**Table 1:** Silicon phthalocyanines and their IC_50_ values^a^

Test compounds	IC_50_ (μM)	
	hCA I	hCA II
ZM-1-Si	0.1653	0.1212
ZM-1-SiQ	0.0710	0.0544
ZM-5-Si	0.0840	0.0762
ZM-5-SiQ	0.0243	0.0363
ZT-Si	0.0178	0.0172
ZT-SiQ	0.0223	0.0260
AZA (Acetazolamide)^35^	0.9857	0.4894

a Errors in the range of 2–5% of the shown data, from three different assays.

We report here the first study on the inhibitory effects of ZM-1-Si, ZM-1-SiQ, ZM-5-Si, ZM-5-SiQ, ZT-Si and ZT-SiQ on the esterase activity of hCA I and II. Data of Table 1 show the following regarding inhibition of hCA I and II with these compounds, by an esterase assay,^36^ with 4-nitrophenylacetate (4-NPA) as substrate:

(i) Against the slow cytosolic isozyme hCA I were moderately inhibited by compound ZM-1-Si. A second group of derivatives, including ZM-5-Si, ZM-1-SiQ and ZM-5-SiQ showed better inhibitory activity as compared to the previously mentioned phthalocyanine, with IC_50_ values in the range of 0.0243–0.0840 μM. Molecules ZT-SiQ and ZT-Si were among the best inhibitors in this series of phthalocyanines. Data of Table 1 also show that similarly to acetazolamide (AZA), some of the investigated phthalocyanines bind in the same regions of the active site cavity as the substrate. However the binding site of 4-NPA itself is unknown, but it is presumed to be in the same region as that of CO_2_, the physiological substrate of this enzyme.^28^

(ii) A rather similar activity of these compounds has been observed also for the inhibition of the rapid cytosolic isozyme, hCA II (Table 1). Thus, a first group of derivatives, ZM-1-Si showed modest hCA II inhibitory activity with IC_50_ in the range of 0.1212 μM (Table 1), whereas the remaining five phthalocyanines, that is, the same compounds acting as efficient hCA II inhibitors, showed IC_50_ in the range of 0.0172–0.0762 μM. The best hCA II inhibitor in this series of derivatives were ZT-Si and ZT-SiQ, which with a IC_50_ of 0.0172–0.026 μM. Considering the data of Table 1, structure–activity relationship was thus quite similar in these small groups of *N*,*N*-dimethylaniline derivatives (phthalocyanines), for both the inhibition of hCA I and II, although differences of affinity between the two isozymes are evident. The *N*,*N*-dimethylamino substituent on phenyl ring could easily be predicted to be involved in making hydrogen bonds with the active site as observed in classical CAI sulfonamide inhibitors (Fig. 1F). Again most of these compounds acted as competitive inhibitors with 4-NPA as substrate (Table 1). The new compounds ZT-Si and ZT-SiQ showed promising powerful inhibitory profiles compared to the standard drug AZA and they all had comparable IC_50_ values against hCA I and hCA II.

In a recent study it was reported that different phenolic compounds,^37^ a simple compound lacking the sulfonamide, sulfamate, or related functional groups that are typically found in all known CA inhibitors, acts as a CAI inhibitor, and could represent the starting point for a new class of inhibitors that may have advantages for patients with sulfonamide allergies.^38^ However, it is critically important to explore further classes of potent CAIs in order to detect compounds with a different inhibition profile as compared to the sulfonamides and their bioisosteres and to find novel applications for the inhibitors of these widespread enzymes.

## 4. Conclusion

In the presented work, novel silicon(iv) phthalocyanine axially substituted with [(2*E*)-3-[4-(dimethylamino)phenyl]-1-(4-phenoxy)prop-2-en-1-one] groups ZT-Si and its quaternized derivative ZT-SiQ were synthesized for the first time. A novel class of efficient CAIs, interacting with the CA isozymes I and II (cytosolic) in a different manner compared to sulfonamides, sulfamates and other classes of inhibitors, is reported in this paper. Kinetic measurements allowed us to identify *N*,*N*-dimethylaniline substituted phthalocyanines as well as ZT-SiQ as submicromolar–low micromolar inhibitors of the two CA isozymes. This new class of inhibitors binds differently of all other CAIs known to date, being found between the phenol-binding site within the enzyme cavity. They exploit different interactions with amino acid residues and water molecules from the CA active site compared to other classes of inhibitors, offering the possibility to design compounds with a better inhibition profile compared to the clinically used sulfonamides/sulfamates. As a result, this study is the first example of carbonic anhydrase enzyme inhibition of phthalocyanines. These results showed that silicon phthalocyanines have potential as carbonic anhydrase inhibitors.

## Conflicts of interest

There are no conflicts to declare.

## Supplementary Material
